# Supplementary material for: Ordering of room-temperature magnetic skyrmions in a polar van der Waals magnet
Source: Nat Commun. 2023 Jun 23;14:3744. doi: 10.1038/s41467-023-39442-0 (PMC10290156; doi:10.1038/s41467-023-39442-0)
Supplement: Supplementary file 1 — Supplementary Information [file 41467_2023_39442_MOESM1_ESM.pdf]

Supplementary Information for: Ordering of Room-Temperature Magnetic Skyrmions in a Polar van der Waals Magnet.

Table of Figures:

1. Examples of solid, liquid, and hexatic phases
2. LTEM of Néel skyrmions
3. MFM images and structure factors of more complete data set
4. Heating and cooling hysteresis
5. Metastability of the skyrmion lattice under the stray field from the MFM tip
6. Orientational domains with temperature of more complete data set
7. Measurement consistency at different monolayer regions
8. Skyrmion order with changing simulation cell size
9. Simulated time stabilization of the skyrmion phase
10. Simulated skyrmions in the bilayer system
11. Regions in the bilayer flake
12. Consistency in bilayer samples

Supplementary Figures

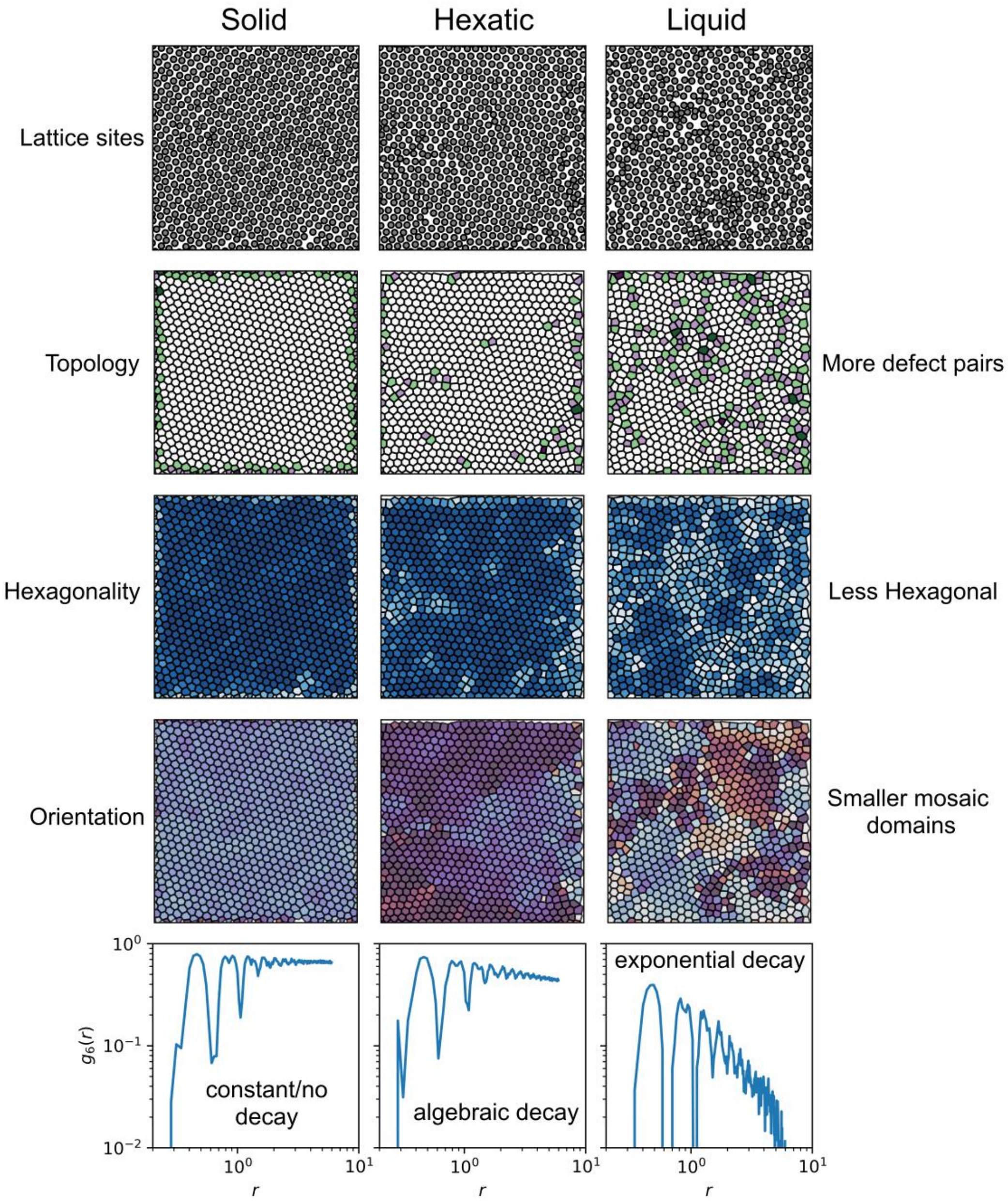

Sup. Figure 1 | Examples of solid, hexatic, and liquid phases. From top to bottom, real space images, nearest neighbor maps, $|\Psi_6|$ maps, Euler angle maps, and $g_6(r)$ showing skyrmion sites, topological defects, hexagonality, mosaic domains, and long-range orientational correlation, respectively.

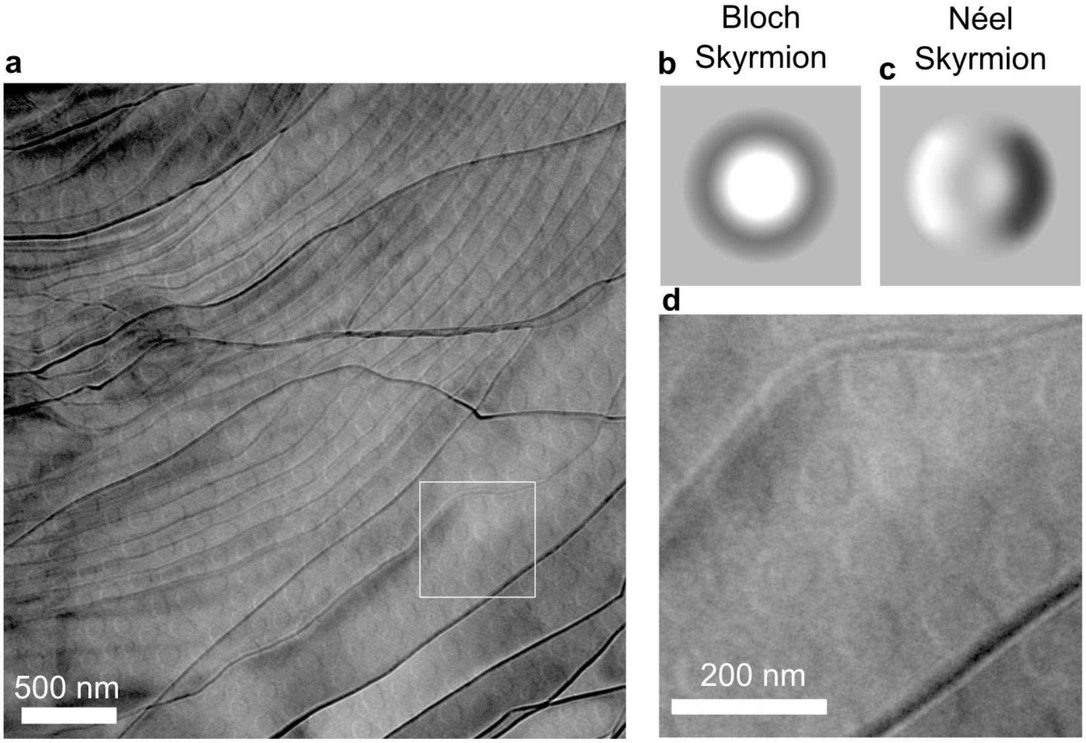

Sup. Figure S2 | LTEM of Néel skyrmions. a Lorentz TEM (LTEM) images of Néel skyrmions and crystallographic defects taken under a sample tilt of 18° and 0 magnetic field. **b** Illustrations of the differences between Bloch and, **c**, Néel skyrmions. **d** Zoomed-in image of the boxed area in a, showing individual skyrmions arranged in hexagonal packing.

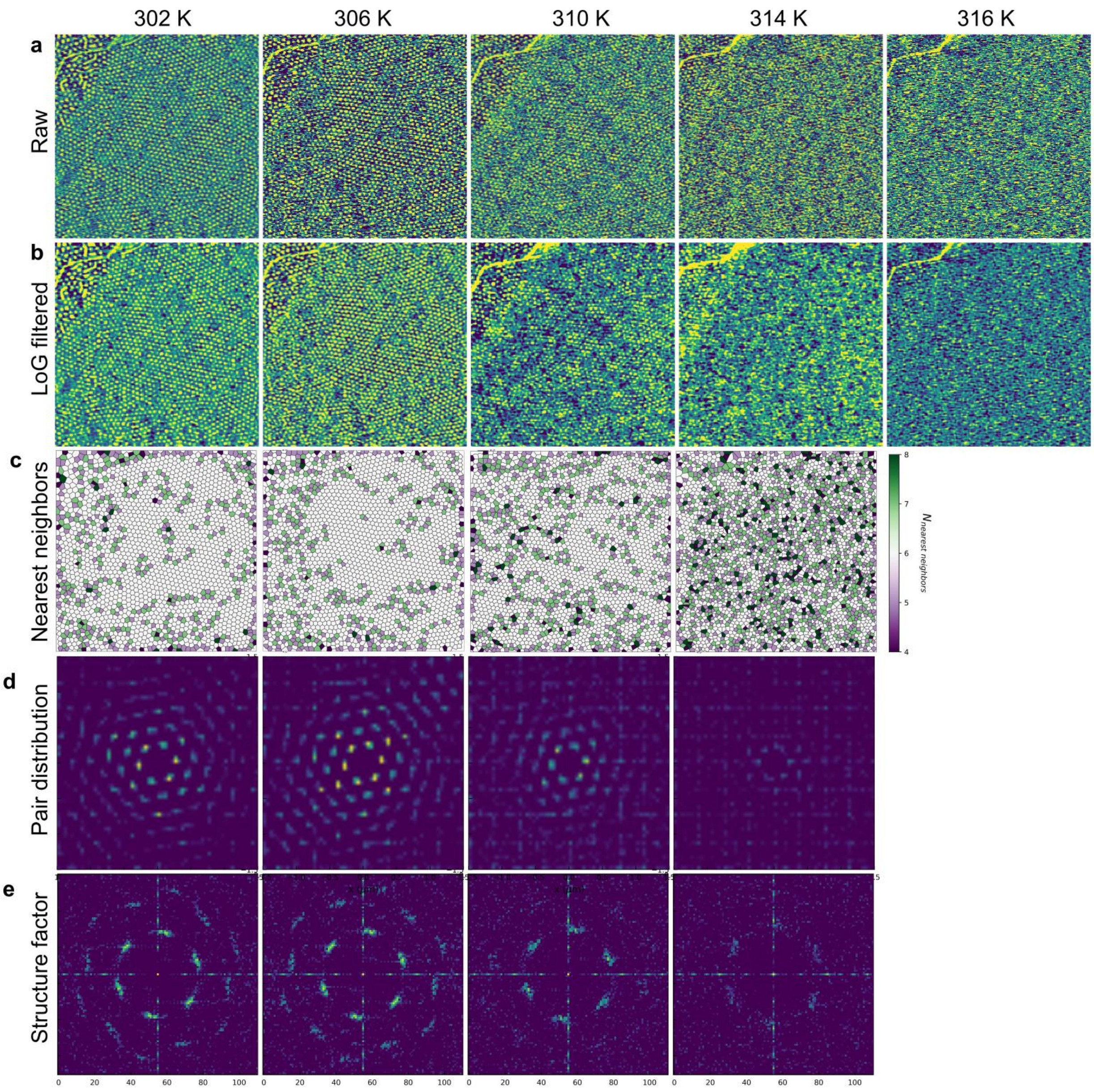

Sup. Figure 3 | MFM images and structure factors. a, Raw, and **b**, Laplacian-of-Gaussian filtered MFM images of skyrmion lattices as a function of temperature. Though the measured T_c is ~350 K, we see the skyrmion crystal phase disappear above 314 K. **c**, the corresponding topology, **d**, pair distribution function, and **e**, structure factors from the images in b. We see that, as temperature increases, the number of topological defects increases, the pair distribution falls off more quickly, and the structure factor changes from being approximately hexagonal, to more circular and diffuse.

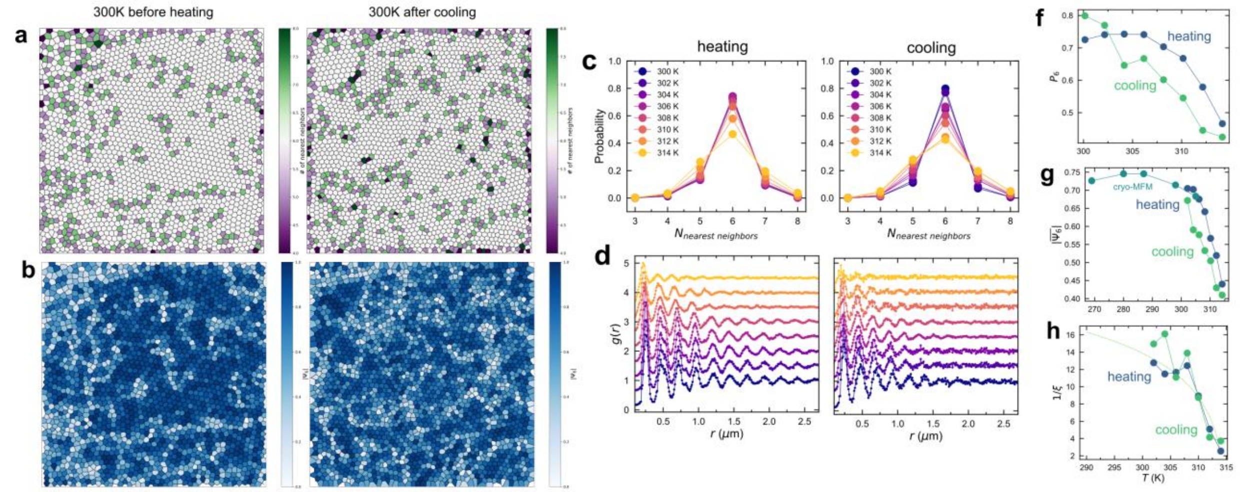

Sup. Figure 4 | Heating and cooling. Nearest neighbor, a, and bond orientational, b, maps of samples on both heating and cooling. **c** Histogram of nearest neighbor probabilities and, **d**, radial distribution function as a function of both cooling and heating. 6-nearest-neighbor probability, P_6 (**f**), mean $|\Psi_6|$ (**g**), and $g_6(r)$ decay $1/\xi$ (**h**) all as functions of temperature, all showing minimal hysteresis between cooling and heating.

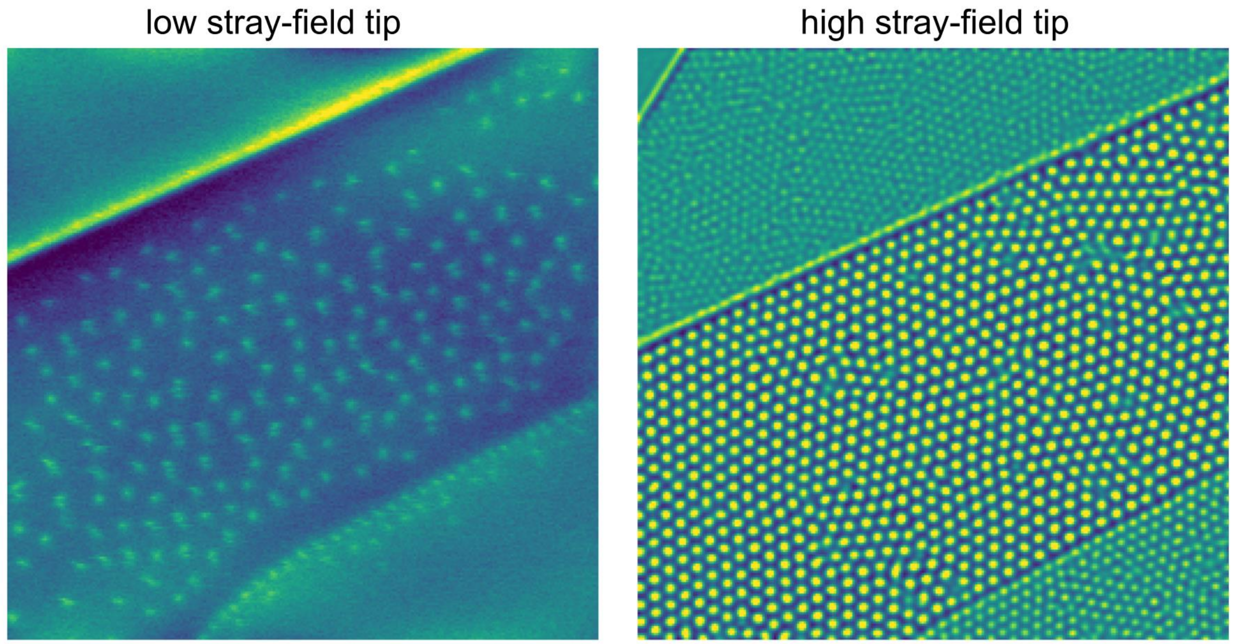

Sup. Figure 5 | Metastability of the skyrmion lattice. From the left image, when the sample is cooled down below T_C, the skyrmions are trapped into a low density, metastable configuration. We then perturb this with the stray field from the MFM tip, to produce the lower energy, high density skyrmion lattice. These results agree with those reported in ref. [2].

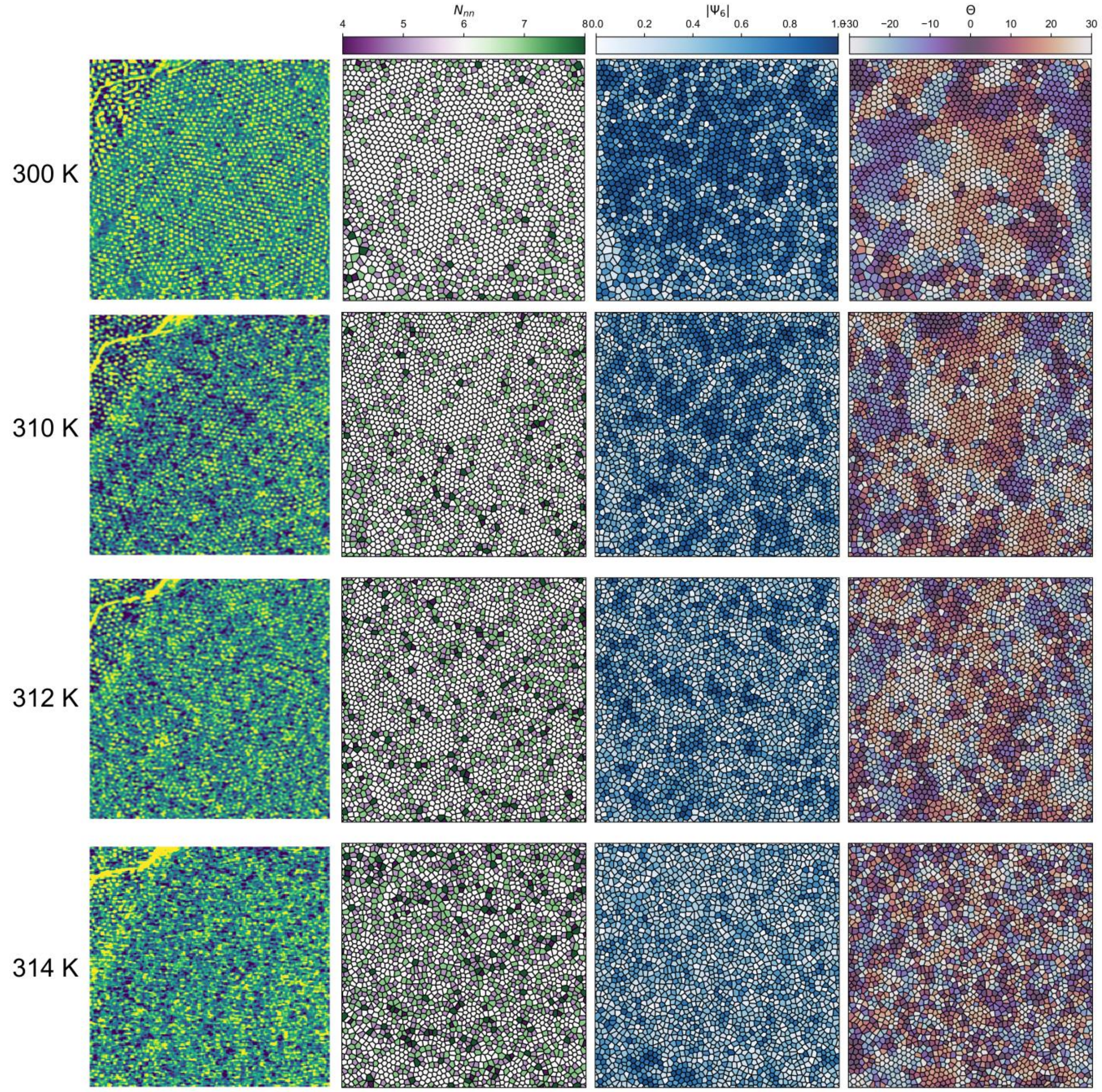

Sup. Figure 6 | Orientational domains with temperature. Real space MFM images, nearest neighbor Voronoi maps, bond orientational maps, and Euler angle maps showing several different temperatures through the phase transition. In the last column, the large domains that are present at low temperatures break up and become less coherent as the lattice melts.

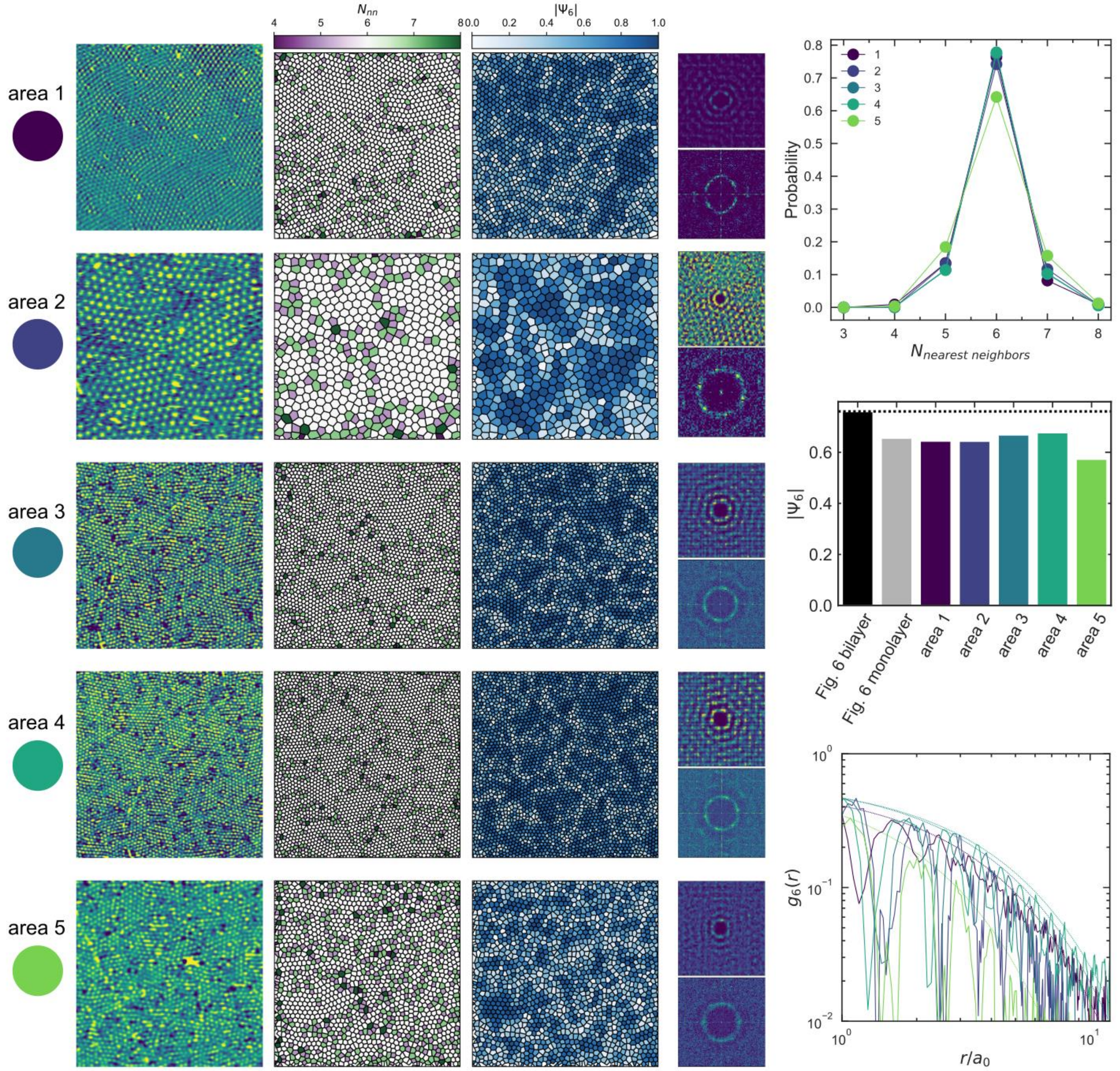

Sup. Figure 7 | Measurement consistency. MFM images, Voronoi maps, Ψ_6 maps, structure factors (SF) and pair distribution functions (PDF) of several other monolayer flake samples and locations, showing that the observed trends hold. In all cases, the monolayer flakes show liquid-like behavior and the $|\Psi_6|$ is in a regime below that of the confined bilayer. Samples 1 and 2 are 143 nm thick, samples 3 and 4 are 185 nm thick, and sample 5 is 207 nm thick.

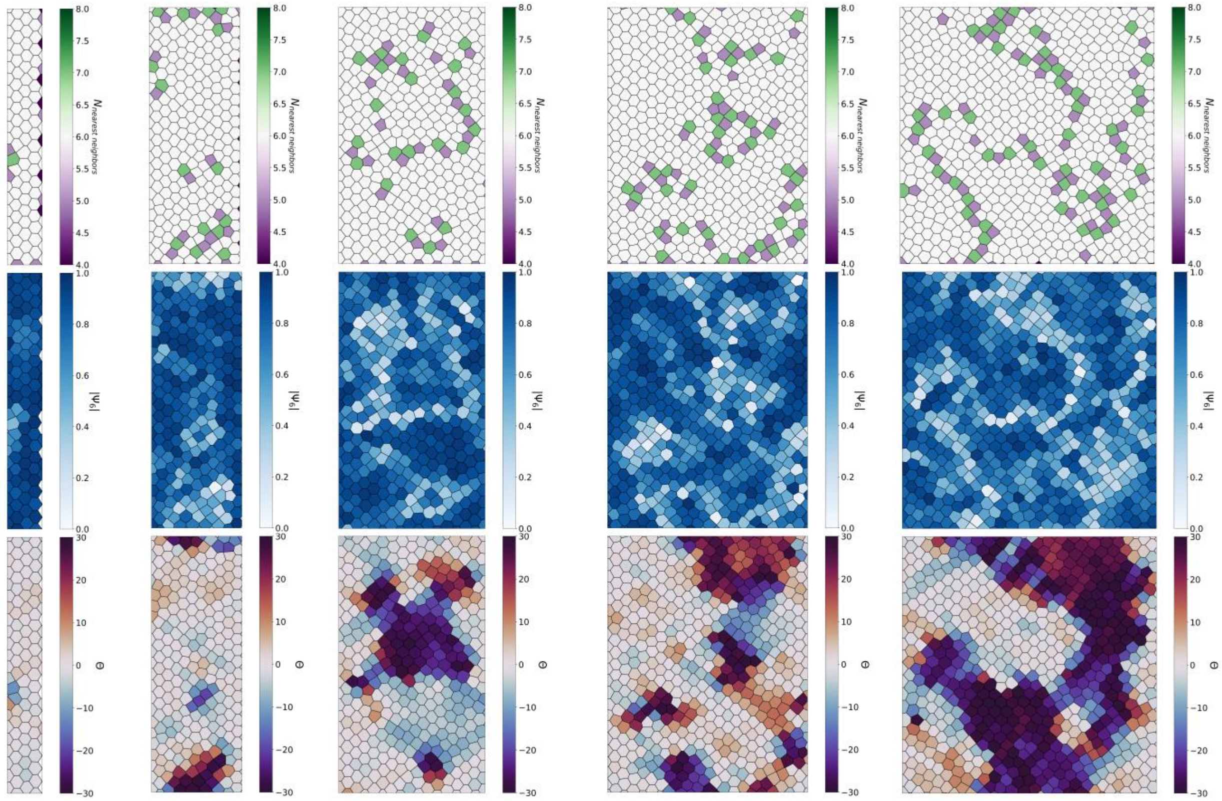

Sup. Figure 8 | Skyrmion order with changing cell size. Increasing the aspect ratio of the simulated cell from 1:5 to 1:1, we see that the skyrmions become qualitatively less ordered. In magnetic skyrmions, interactions with the sample edge are likely due to magnetoelastic or shape anisotropies, which we suspect is also the case in our system. In the steady state, we would expect an ordering of the skyrmions at the edge to minimize stray magnetic field/interaction with the large potential at the boundary (e.g. shape anisotropy), favoring a locally ordered configuration.

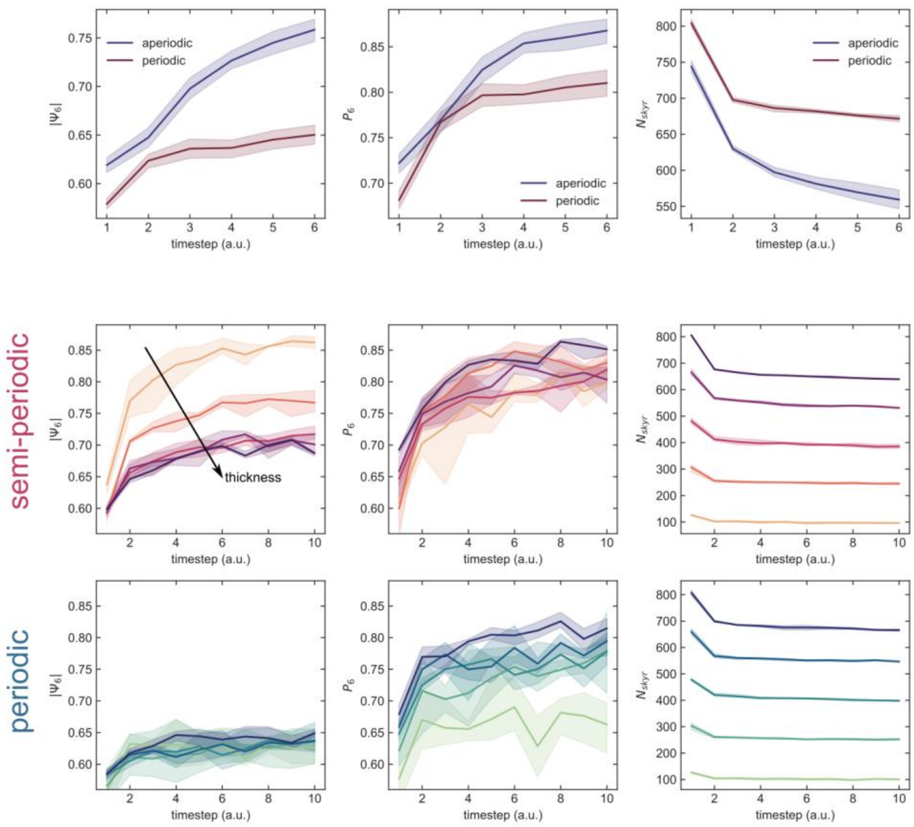

Sup. Figure 9 | Simulated stabilization of the skyrmion phase. Order parameters showing the evolution of the skyrmion phase as a function of arbitrary timesteps in the simulation. Also highlighted, are the differences between different aspect ratios in the semi-periodic case. The arrow shows increasing cell width corresponding to the plots in Sup. Figure 6.

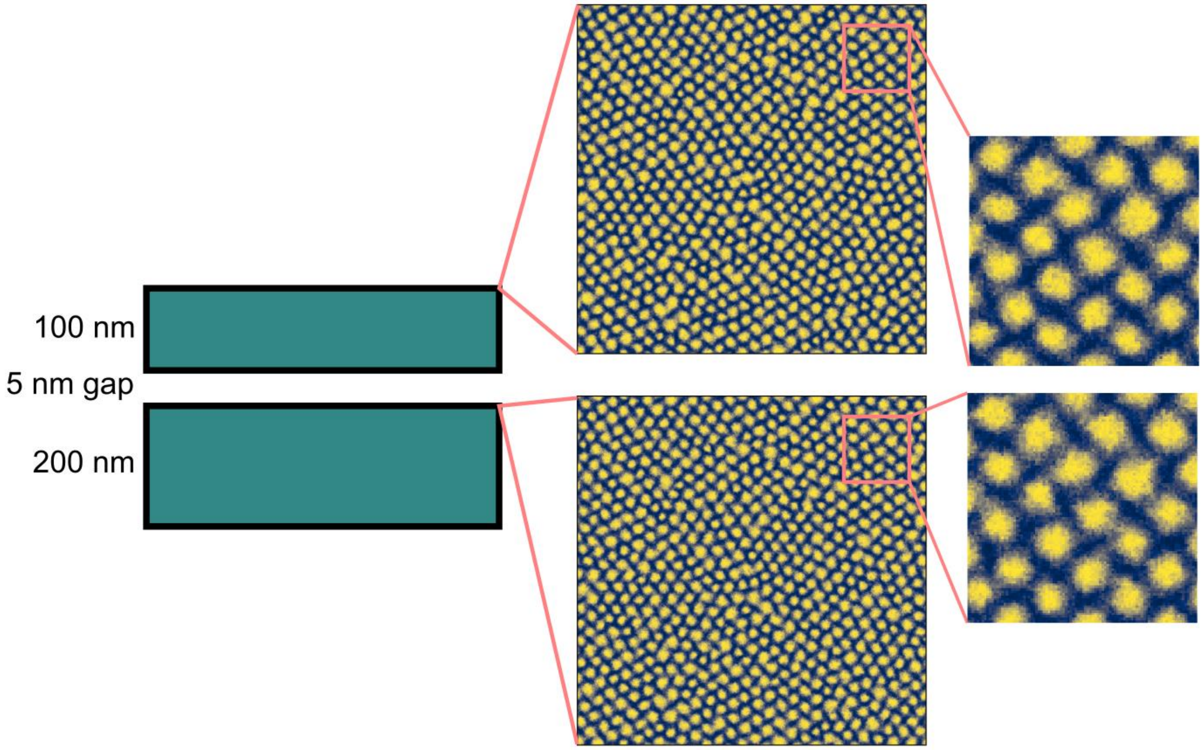

Sup. Figure 10 | Skyrmions in the bilayer system. Simulation showing stable skyrmions in the bilayer system with a 5 nm gap between the layers, where skyrmions track from one layer to the other, indicating that the bilayer behaves as a single magnet.

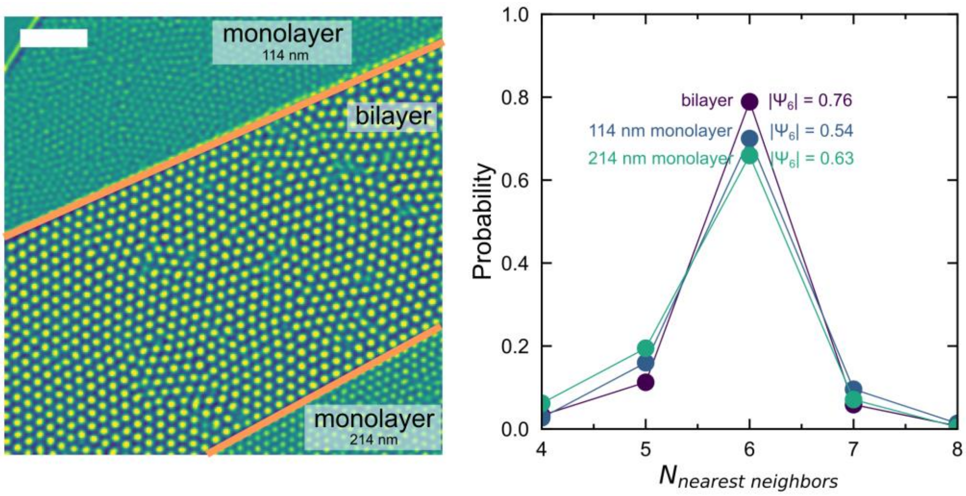

Sup. Figure 11 | Regions of the bilayer flake. MFM image showing monolayer and bilayer FCGT regions with the corresponding histogram for N_{nn} and $|\Psi_6|$ values. These values indicate a disordered phase in the lower-right 214 nm monolayer region, which is

consistent with the explicitly disordered top left 114 nm region, but the statistics cannot be rigorously defined because of the small population size.

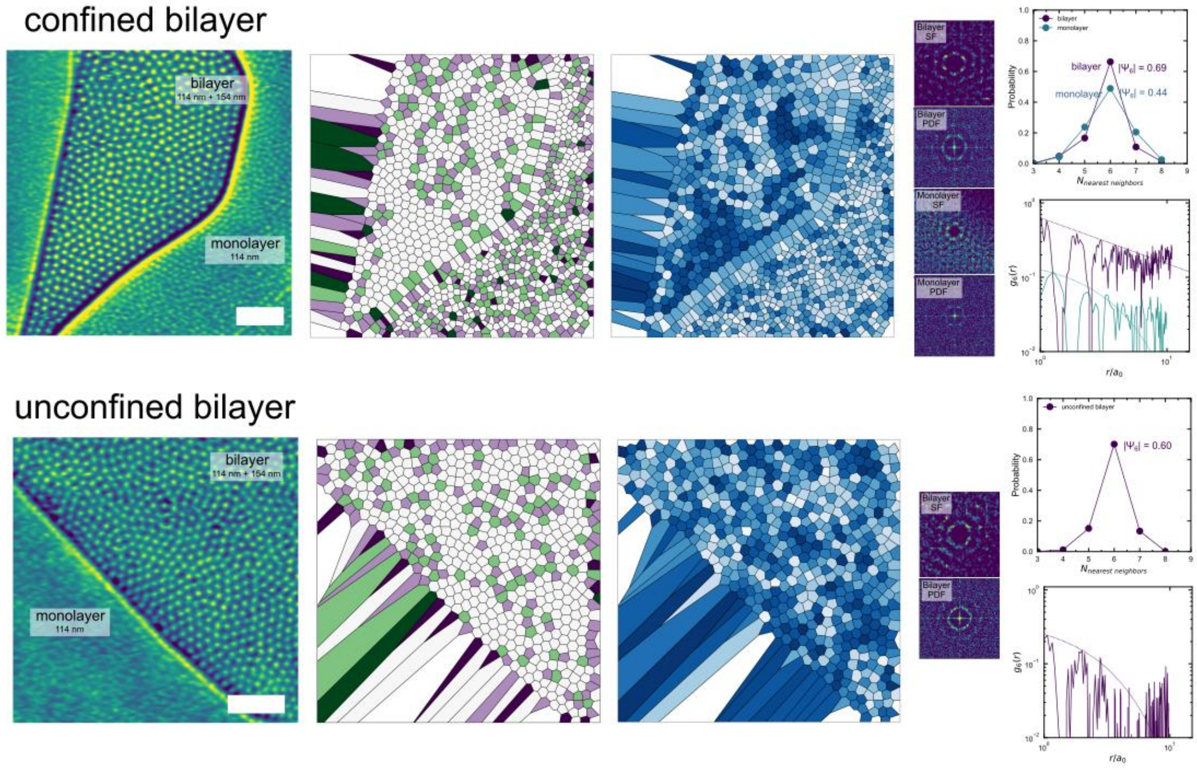

Sup. Figure 12 | Consistency in bilayer samples. MFM images, Voronoi maps, Ψ_6 maps, structure factors (SF) and pair distribution functions (PDF) of other bilayer samples and locations, showing that the observed trends hold. Samples show liquid-like behavior when the areas are unconfined, such as in monolayer flakes and bilayer flakes only bounded on one side, and ordered hexatic-like behavior when bilayer regions are bounded on both sides. Qualitatively, we observe that skyrmions always order along the edge of the flake, supporting our assertion that the ordering field from the confinement is trapping or nucleating skyrmions, which increase the stability of nearby structural domains.

References

1. Huang, P. *et al.* Melting of a skyrmion lattice to a skyrmion liquid via a hexatic phase. *Nat. Nanotechnol.* **15**, 761–767 (2020).
2. Zhang, H. *et al.* Room-temperature skyrmion lattice in a layered magnet (Fe0.5Co0.5)5GeTe2. *Science Advances* **8**, eabm7103 (2022).
